# 1,25-Dihydroxyvitamin D_3_ Suppresses Prognostic Survival Biomarkers Associated with Cell Cycle and Actin Organization in a Non-Malignant African American Prostate Cell Line

**DOI:** 10.3390/biology13050346

**Published:** 2024-05-15

**Authors:** Jabril R. Johnson, Rachel N. Martini, Yate-Ching Yuan, Leanne Woods-Burnham, Mya Walker, Greisha L. Ortiz-Hernandez, Firas Kobeissy, Dorothy Galloway, Amani Gaddy, Chidinma Oguejiofor, Blake Allen, Deyana Lewis, Melissa B. Davis, K. Sean Kimbro, Clayton C. Yates, Adam B. Murphy, Rick A. Kittles

**Affiliations:** 1 Department of Microbiology, Biochemistry, & Immunology, Morehouse School of Medicine, 720 Westview Drive, Atlanta, GA 30310, USA; 2 Institute of Translational Genomic Medicine, Morehouse School of Medicine, 720 Westview Drive, Atlanta, GA 30310, USA; 3 Department of Computational Quantitative Medicine, Center for Informatics, City of Hope Comprehensive Cancer Center, Duarte, CA 91010, USA; 4 Department of Physiology, Morehouse School of Medicine, 720 Westview Drive, Atlanta, GA 30310, USA; 5 Department of Diabetes and Cancer Metabolism, Beckman Research Institute, City of Hope Comprehensive Cancer Center, Duarte, CA 91010, USA; 6 Department of Population Sciences, Beckman Research Institute, City of Hope Comprehensive Cancer Center, Duarte, CA 91010, USA; 7 Department of Neurobiology, Center for Neurotrauma, Multiomics & Biomarkers (CNMB), Neuroscience Institute, Morehouse School of Medicine, 720 Westview Dr SW, Atlanta, GA 30310, USA; 8 Department of Community Health and Preventive Medicine, Morehouse School of Medicine, 720 Westview Drive, Atlanta, GA 30310, USA; 9 Department of Urology, Johns Hopkins University School of Medicine, Baltimore, MD 21205, USA; 10 Department of Pathology, Johns Hopkins University School of Medicine, Baltimore, MD 21205, USA; 11 Department of Urology, Feinberg School of Medicine, Northwestern University, Chicago, IL 60611, USA

**Keywords:** vitamin D, calcitriol, prostate cancer disparities, actin cytoskeleton, cell cycle, RNA-Seq, non-malignant prostate cell, *ANLN*, *ECT2*, Rho GTPase

## Abstract

**Simple Summary:**

Vitamin D_3_ is a steroid hormone that has been shown to prevent tumor growth in prostate cells. Not having enough vitamin D_3_ in the blood has been linked to advanced prostate cancer and mortality, especially in African American men. We wanted to understand how vitamin D affected pathways that keep prostate cells from becoming cancerous, which could lead to new therapeutic targets and treatments, especially for African American men who tend to be more prone to being vitamin D deficient compared to European men. Here, we studied a non-cancerous African American prostate cell line treated with the active form of vitamin D with a concentration similar to what is found in the body for 24 h. Using RNA whole-transcriptome sequencing, we compared these treated cells with untreated cells to assess genes and pathways significantly changed due to treatment. We found that vitamin D affected the activity of 1601 genes, mainly suppressing pathways linked to prostate cell movement, growth, and viability. Only two genes, *ANLN* and *ECT2*, were strongly correlated with prostate cancer prognosis and patients tended to have better survival rates when these genes were less active. Furthermore, downregulation of *ANLN* and *ECT2* was also shown to repress signaling pathways involved in prostate cell movement, growth, malignant transformation, and viability. Our results suggest that vitamin D decreases the activity of these genes and could be important for preventing prostate cancer, especially for African American men. This could lead to the development of new treatments targeting specific genes and pathways involved in prostate cancer growth.

**Abstract:**

Vitamin D_3_ is a steroid hormone that confers anti-tumorigenic properties in prostate cells. Serum vitamin D_3_ deficiency has been associated with advanced prostate cancer (PCa), particularly affecting African American (AA) men. Therefore, elucidating the pleiotropic effects of vitamin D on signaling pathways, essential to maintaining non-malignancy, may provide additional drug targets to mitigate disparate outcomes for men with PCa, especially AA men. We conducted RNA sequencing on an AA non-malignant prostate cell line, RC-77N/E, comparing untreated cells to those treated with 10 nM of vitamin D_3_ metabolite, 1α,25(OH)_2_D_3_, at 24 h. Differential gene expression analysis revealed 1601 significant genes affected by 1α,25(OH)_2_D_3_ treatment. Pathway enrichment analysis predicted 1α,25(OH)_2_D_3-_ mediated repression of prostate cancer, cell proliferation, actin cytoskeletal, and actin-related signaling pathways (*p* < 0.05). Prioritizing genes with vitamin D response elements and associating expression levels with overall survival (OS) in The Cancer Genome Atlas Prostate Adenocarcinoma (TCGA PRAD) cohort, we identified *ANLN* (Anillin) and *ECT2* (Epithelial Cell Transforming 2) as potential prognostic PCa biomarkers. Both genes were strongly correlated and significantly downregulated by 1α,25(OH)_2_D_3_ treatment, where low expression was statistically associated with better overall survival outcomes in the TCGA PRAD public cohort. Increased *ANLN* and *ECT2* mRNA gene expression was significantly associated with PCa, and Gleason scores using both the TCGA cohort (*p* < 0.05) and an AA non-malignant/tumor-matched cohort. Our findings suggest 1α,25(OH)_2_D_3_ regulation of these biomarkers may be significant for PCa prevention. In addition, 1α,25(OH)_2_D_3_ could be used as an adjuvant treatment targeting actin cytoskeleton signaling and actin cytoskeleton-related signaling pathways, particularly among AA men.

## 1. Introduction

Prostate cancer (PCa) is the second most frequently diagnosed malignancy and the second leading cause of death in men in the United States (U.S.), after adjusting for age. African Americans (AAs) have a 1.7 increased incidence and 2.4 times higher mortality rate compared to European Americans (EAs) [1]. In addition, AA men often present to the clinic with higher serum levels of Prostate-Specific Antigen (PSA) and high-grade tumors than EA men, even in equal access healthcare systems [2]. The factors contributing to the glaring disparities in incidence and outcome are multifaceted and include sociocultural (e.g., screening, recent smoking, medical mistrust), socio-political (e.g., neighborhood and individual socioeconomic status and access to healthcare), and biological (e.g., genetic risk and vitamin D deficiency) variables [3]. Yet, even after adjusting for the effects of non-biological determinant factors, racial disparities in mortality rates remain significant [3,4,5,6]. This suggests that biological determinants may contribute greatly to PCa disparities in AA men [3,7,8,9]. 

One potential driver of AA PCa arises from vitamin D deficiency, which alters vitamin D_3_ signaling [10]. Epidemiological studies assessing the impact of vitamin D and/or its metabolites on PCa progression have shown that deficient serum vitamin D_3_ levels are associated with higher PCa stage, grade, and mortality in AA men [11,12,13]. In addition, serum vitamin D deficiency was associated with increased odds of PCa diagnosis on biopsy in AA men [14]. African American men are also more likely to have deficient serum vitamin D_3_ levels compared to EA men [13,15]. However, serum vitamin D deficiency association with PCa risk remains inconclusive. For example, in a randomized controlled study, the vitamin D and omega-3 trial (VITAL) investigated the efficacy of vitamin D supplementation as primary prevention of cancer within 5 years. After 5 years of follow-up, the vitamin D and omega-3 group had the same overall cancer incidence as the placebo group. Conversely, in this study, PCa incidence among AA men was reduced by 23% (*p* < 0.07) [16], which suggests further investigation is needed to elucidate the relationship between serum vitamin D levels’ and PCa risk reduction in high-risk populations (e.g., AA men).

Many pre-clinical studies have been consistent in their demonstration of malignant cells eliciting an anti-tumorigenic response upon exposure to physiological (10 nM) or higher concentrations of the active form of vitamin D, 1α,25(OH)_2_D_3_ [17,18,19,20]. For example, 100 nM of 1α,25(OH)_2_D_3_ was shown to inhibit cellular proliferation in endometrial carcinoma cells by targeting and modulating actin cytoskeleton organization via the ras-related C3 botulinum toxin substrate 1 (*RAC1*)/p21 protein-activated kinase 1 (*PAK1*) signaling pathway [21,22]. In PCa, Cyclooxygenase-2 (*COX*-2) is overexpressed in putative cancer precursor inflammatory lesions, tumor-infiltrating macrophages, and other cells in the microenvironment of prostates [23]. Data suggest 10 nM and 100 nM of 1α,25(OH)_2_D_3_ regulate the expression of several genes in the prostaglandin pathway, thus inhibiting growth of PCa cells, in vitro and in vivo [23]. However, despite consistent data demonstrating varying concentrations of 1α,25(OH)_2_D_3_ eliciting anti-tumorigenic effects in different pre-clinical models, it is important to note that increased concentrations of 1α,25(OH)_2_D_3_ are also associated with risk of developing hypercalcemia, thus hampering clinical applications [24]. Therefore, elucidating the potential protective effects of physiological concentrations of 1α,25(OH)_2_D_3_, which modify mechanisms preventing prostate carcinogenesis, is imperative, especially in high-risk populations (e.g., AA men).

In this present study, we examined the effect of 10 nM of 1α,25(OH)_2_D_3_ on the transcriptome profile of a non-malignant AA prostate cell line, RC-77N/E. We hypothesized physiological concentrations of 1α,25(OH)_2_D_3_ significantly suppressed genes that are essential to maintaining a non-malignant phenotype in AA prostate cells. Here, we report 1α,25(OH)_2_D_3_ suppresses potential PCa survival biomarkers’, *ANLN* (Anillin) and *ECT2* (Epithelial Cell Transforming 2), expressions in non-malignant AA prostate cells. Furthermore, 1α,25(OH)_2_D_3_ treatment led to the suppression of differentially expressed genes (DEGs) and signaling pathways that underly prostate carcinogenesis and malignant transformation. Thus, elucidating the effects of 1α,25(OH)_2_D_3_ on mechanisms that underly prostate carcinogenesis is crucial to developing novel interventions for PCa prevention and adjuvant treatment, especially for AA patients that may be more prone to being vitamin D deficient [13].

## 2. Materials and Methods

### 2.1. Ancestry Genotyping in Non-Malignant AA Cell Line RC-77N/E

Single nucleotide polymorphisms (SNPs), previously authenticated for determining continental ancestry in mixed populations, were chosen as ancestry informative markers (AIMs) [25,26,27]. The AIM panel comprised 105 SNPs and underwent genotyping via the Sequenom MassARRAY platform utilizing iPLEX chemistry, following the manufacturer’s guidelines. iPLEX assays were formulated using Sequenom Assay Design software (version 4.0) to enable single-base extension designs for multiplexing. 10 ng of genomic DNA, extracted from RC-77N/E underwent PCR (based on manufacturers protocol). Subsequently, shrimp alkaline phosphatase enzyme treatment was applied to PCR reactions to neutralize unincorporated deoxyribonucleotide triphosphate. A post-PCR single-base extension reaction followed each multiplex reaction, utilizing concentrations of 0.625 μmol/L for low-mass primers and 1.25 μmol/L for high-mass primers. The reactions were then diluted with 16 μL of H_2_O, and fragments were purified with resin before being spotted onto Sequenom Spectro CHIP microarrays (Agena Bioscience, San Diego, CA, USA, Product 10500) for scanning by MALDI-TOF mass spectrometry. Individual SNP genotype calls were generated using Sequenom TYPER software (version 4.0), which automatically identified allele-specific peaks based on their expected masses. A 99% concordance rate was observed across all markers for genotypes. Genotyping call rates surpassing 98.5% were integrated into the analyses. Individual admixture estimates were computed utilizing a model-based clustering method within software STRUCTURE v2.3 [28]. The program was executed with k = 3 parental population genotypes representing West Africans, Europeans, and individuals of Native American ancestry [25] under the admixture model using the Bayesian Markov chain Monte Carlo method and a burn-in length of 30,000 for 70,000 repetitions. As the genetic ancestry of the RC-77N/E cell line has not been previously documented, we employed the admixture model to ascertain the optimal estimation of K (number of sub-populations) that best aligns with the data.

### 2.2. Sample Preparation: Cell Culture and 1α,25(OH)_2_D_3_ Treatment

RC-77N/E was grown in a humidified incubator with 5% CO_2_ at 37 °C. In accordance with recent guidelines by the National Institutes of Health (NIH) on the authentication of key biological resources, cells were authenticated utilizing Short Tandem Repeat (STR) profiling against the American Type Culture Collection (ATCC) STR database (ATCC, Cat: ATCC 135-XV). RC-77N/E cells were routinely tested for mycoplasma contamination using MycoProbe Mycoplasma Detection Kit (R&D Systems, Minneapolis, MN, USA, Cat: CUL001B). RC-77N/E cell line was provided by C.A. Casiano’s laboratory with permission from J.S. Rhim. RC-77N/E was grown in collagen-coated treated culture dishes in keratinocyte serum-free medium (K-SFM) supplemented with bovine pituitary extract and recombinant epidermal growth factor (Gibco, Grand Island, NE, USA, Cat:17-005-042) and was used for growing and maintaining the cells. Normocin 0.2% (Invivogen, San Diego, CA, USA, Cat: ANT-NR-1) was added to the medium for all cell lines to prevent contamination by mycoplasma, bacteria, or fungi. For treatment, 1,25-dihydroxyvitamin D_3_ [1α,25(OH)_2_D_3_] (Tocris Bioscience, Bristol, UK, & Cat: 25-515-0U) reconstituted in 100% ethanol was used. Cells were then treated with 10 nM (physiological concentration) for 24 h. Vehicle (ethanol) was included for every untreated control. The final ethanol percentage for treated and untreated (vehicle control) cell lines was 0.01%.

### 2.3. RNA Isolation from RC-77N/E Prostate Cell Line

Before handling RNA, surfaces and instruments underwent treatment with RNase Zap (Ambion AM9780, Austin, TX, USA). RNA extraction from cultured cells followed the Rneasy Mini protocol (Qiagen 7414, Hilden, Germany). Initially, cells were rinsed with PBS, then lysed with buffer RLT and 10 μL/mL β-mercaptoethanol, utilizing 350 μL per T25 flask or 600 μL per 10 cm plate. Subsequently, the Rneasy Mini protocol, inclusive of on-column incubation with Rnase-free Dnase (Qiagen, Cat: 79254), was adhered to. Post washes, RNA was eluted using 30 μL Rnase-free water, and its concentration was determined employing a Nanodrop spectrophotometer. RNA samples were stored at −80 °C, with each sample being isolated and utilized individually in triplicate. All raw data generated in this study, including FASTQ sequencing data and metadata, have been submitted to the NCBI Gene Expression Omnibus (GEO; https://www.ncbi.nlm.nih.gov/geo/ (submitted on 13 May 2024) under accession number GSE267396. https://www.ncbi.nlm.nih.gov/geo/query/acc.cgi?acc=GSE267396.

### 2.4. RNA Quality Control

The purity and concentration of RNA samples were determined from Optical Density 260/280 readings using a dual-beam UV spectrophotometer, and RNA integrity was determined by capillary electrophoresis using the Agilent Bioanalyzer DNA High Sensitivity Kit and quantified with Qubit, as per the manufacturer’s instructions.

### 2.5. RNA Sequencing Library Preparation and Sequencing with Illumina HiSeq 2500

RNA sequencing was performed by the City of Hope Integrative Genomics Core facility using RNA sequencing libraries prepared with Kapa RNA mRNA HyperPrep kit (Kapa Biosystems, Wilmington, NC, USA, Cat KR1352) according to the manufacturer’s protocol. The final libraries were validated with the Agilent Bioanalyzer DNA High Sensitivity Kit and quantified with Qubit. Sequencing was performed on Illumina HiSeq 2500 with the single read mode of 51 cycles. Real-time analysis (RTA) 2.2.38 software was used to process the image analysis.

### 2.6. RNA-Seq Quality Control, Alignment, and Quantification

Quality of the raw RNA-Seq read data was assessed using FastQC (v0.11.7) [29]. Read trimming and adapter removal were performed using Trimmomatic (v0.36) [30], and the quality of trimmed reads was assessed with FastQC. Trimmed reads were aligned to the reference genome (GrCh37) using HISAT2 (v2.1.0) [31]. Gene expression was quantified from alignments using Stringtie (v1.3) [31] and reported in transcripts per million (TPM). 

### 2.7. DEG Analyses

DESeq2 [32] was utilized to compare gene expression between RC-77N/E triplicates (*n* = 3) treated with 1α,25(OH)_2_D_3,_ and untreated control triplicates (*n* = 3). However, due to a treatment sample replicate presenting as an outlier and not passing our QC threshold, it was not used in downstream analysis. The input gene counts matrix was generated using Stringtie (v.1.3). Using an FDR adjusted *p*-value threshold of 0.05, 1601 genes were identified as significantly differentially expressed. Heatmap, Principal Component Analysis (PCA), and volcano plot visualization were generated using JMP Pro 16.0.0 (SAS Institute Inc., Cary, NC, USA, 2989-2022).

### 2.8. DEG Selection Algorithm

DEGs of interest were chosen utilizing a DEG selection algorithm. Our algorithm is as follows: DEGs, generated via RNA–Seq, were filtered by a significance threshold of FDR < 0.05. DEGs of interest were further filtered using ChIP–Seq public datasets for screening. DEGs within public datasets were differentially regulated by vitamin D analogues and their putative vitamin D response elements (VDRE) motifs were identified. Kaplan–Meier survival analysis was used to screen for DEGs associated with PCa patient survival. Lastly, DEGs were validated via RT-qPCR and PCa cohorts.

### 2.9. DEG Association with Survival of PCa Patients Using the Cancer Genome Atlas Prostate Adenocarcinoma (TCGA PRAD) Cohort

To identify DEGs generated from our RNA-Seq analysis that were associated with survival of PCa patients, we performed a Kaplan–Meier survival analysis utilizing the TCGA PRAD cohort. Whole-transcriptome sequences of 542 PCa patients were downloaded from TCGA PRAD cohort (https://portal.gdc.cancer.gov/projects/TCGA-PRAD, accessed on 1 October 2022). The “high” and “low” groups were segregated based on median mRNA expression values. Kaplan–Meier survival analysis was used to determine the survival differences between “high” and “low” mRNA expression groups, which were visualized by Kaplan–Meier plots and compared using Cox regression analysis, with *p*-values calculated by log-rank test using the survival package in R (version 3.6-4) [33]. The survival differences were statistically significant when *p*-values were <0.05. 

### 2.10. In Silico Bioinformatic Analysis of DEGs in 1α,25(OH)_2_D_3_-Treated RC-77N/E Cells

Pathway enrichment analyses were completed using Ingenuity Pathway Analysis ((IPA) v73620684) (2022 QIAGEN Inc., Redwood City, CA, USA, https://digitalinsights.qiagen.com/IPA, accessed on 20 May 2022) [34]. Of the 1601 DEGs, 33 DEGs were not mapped and subsequently not added to pathway analysis. According to IPA, unmapped genes may result from the following 3 scenarios: (1) the gene/protein ID does not correspond to a known gene product; (2) the identifier is associated with more than one Entrez Gene ID and is, therefore, left unmapped in the application due to the ambiguity of its identity; and/or (3) the identifier format may be shared by multiple sources (for example, Entrez Gene and PubChem both use integer values). Enrichment analysis with FDR *p*-value < 0.05 was reported. Z-scores represent predicted activation (Z > 0, orange) or inhibition (Z < 0, blue) based on log2 fold change values, and known interactions from the IPA Knowledge Base. Gene ontology (GO) analysis, with up- and downregulated enrichment results for complete biological processes, molecular functions, and cellular component categories (Benjamini–Hochberg (B–H) corrected q value < 0.05), was performed using Database for Annotation, Visualization, and Integrated Discovery (DAVID) (https://david.ncifcrf.gov/summary.jsp, accessed on 10 June 2022) [35,36]. 

Furthermore, we conducted both an interactome and pathway analysis interrogation utilizing Elsevier’s Pathway Studio version 10.001 (https://www.elsevier.com/solutions/pathway-studio-biological-research, accessed on 1 September 2023), and STRING pathway analysis. To delineate the relationships among the differentially expressed genes (DEGs) pertinent to PCa, we employed the ResNet Pathways Studio Proprietary database, which comprises relationships between proteins compiled from gene ontology and the PubMed literature. The gene/proteome interactome network was generated using a “direct interaction” algorithm for cellular and biological process mapping, alongside proposed pathway interactions. 

Fisher’s statistical test within the Pathway Studio tool was employed to discern nonrandom associations between specific relationships (protein interaction and biological process). Subsequently, a one-sided Mann–Whitney U test compared the sub-network distribution to the background distribution, yielding *p*-values indicative of statistical significance. Our analysis entailed the importation of “GenBank ID” and gene symbols to form an experimental dataset, wherein we selected for inclusion all *p*-values less than 0.005 (biological process) and overlapping genes greater than 2.

The supplementary information contains the raw data of pathways selected through statistical analysis (*p*-value < 0.005). These data encompass gene ID, references used in establishing relationships, interaction types (binding, post-translational modification, modulation, inhibition, etc.), and associated *p*-values for each gene–gene interaction and gene–molecular function interaction. Furthermore, STRING (version 12.0) was utilized to evaluate protein–protein network interactions and predict co-expression of significant DEGs identified from our RNA-Seq analysis with genes of interest, namely, focusing on *ANLN* and *ECT2*. Significance was ascertained using a *p*-value threshold of <0.05 [37]. 

### 2.11. Validation of RNA-Seq Data through RT-qPCR

To quantitatively assess the reliability of our sequencing data, the expression levels of our 2 identified targets and 3 randomly relevant selected genes (*n* = 5) were analyzed with RT-qPCR in the same samples used for RNA-Seq. Total RNA was extracted from cells using the PureLink RNA Mini Kit (Thermo Fisher Scientific, Waltham, MA, USA, Cat. 12183025). RNA yield and quality were determined by the absorbance ratio at 260/280 nm using the NanoDrop One Microvolume UV–Vis Spectrophotometer (Thermo Fisher Scientific, Waltham, MA, USA, Cat. ND-ONE-W). Total RNA (500 ng) was reverse transcribed into cDNA using the High-Capacity RNA-to-cDNA kit (Thermo Fisher Scientific, Waltham, MA, USA, Cat. 4387406) and random hexamer primers following the manufacturer’s protocol. RT-qPCR was performed using the PowerUp SYBR Green Master Mix (Thermo Fisher Scientific, Waltham, MA, USA, Cat. A25742) following the manufacturer’s fast cycling mode protocol. Primers were designed using Primer3Plus software (version 3.3.0) and commercially synthesized by Integrated DNA Technologies (IDTs). cDNAs were amplified using *ECT2* Forward: 5′-CATGTCGCCCGTTGTATTGT-3′, Reverse: 5′-AGACTCACAGCAACCCTGAA-3′; ANLN Forward: 5′-GGTGTGGTAAGTCCAGAGAGTT-3′, Reverse: 5′-CACCAGATTCAGCTCGAGGG-3′; *IL7R* Forward: 5′-GGATCGCAGCACTCACTGAC-3′, Reverse: 5′-GGCACTTTACCTCCACGAGGG-3′; *IL8* Forward: 5′-GCAGTTTTGCCAAGGAGTGCT-3′, Reverse: 5′-CCTTGGGGTCCAGACAGAGC-3′; *GDF15* Forward: 5′-TGCGGAAACGCTACGAGGAC -3′, Reverse: 5′- GCCCGAGAGATACGCAGGTC; and hGAPDH Forward: 5′-TCGGAGTCAACGGATTTGGT-3′; Reverse: 5′-TTCCCGTTCTCAGCCTTGAC-3′. RT-qPCR amplification was performed using the QuantStudio 3 Real-Time PCR System (Thermo Fisher Scientific, Waltham, MA, USA, Cat. A28137). Reactions were performed with biological replicates, and data were normalized to the reference gene *GAPDH*. Fold change in target gene expression treated cells relative to the untreated controls was calculated using the ΔΔCT method.

### 2.12. Validation of Selected DEGs in 1α,25(OH)_2_D_3_-Treated Non-Malignant EA Prostate Cell Line, RWPE1

To further explore 1α,25(OH)_2_D_3_ suppression of *ANLN* and *ECT2* gene expression, that was identified in our cell line analyses, we utilized public Affymetrix U133 Plus 2.0 mRNA gene expression profile published using RWPE1, a non-tumorigenic European American prostate epithelial cell line, treated with 1α,25(OH)_2_D_3_ (*n* = 4/treatment at 6 h, 24 h, 48 h using 100 nM dosage). The processed microarray data were downloaded at the NCBI Gene Expression Omnibus (GEO) database (accession #GSE15947). The differential gene expression and correlation analyses were performed using OmicSoft Studio software version 12.2. within OncoGEO B37 (QIAGEN^®^, Redwood City, CA, USA) with *p*-value < 0.05 reported.

### 2.13. Validation of DEGs in Prostate Clinical Cohorts 

*ANLN* and *ECT2* expression levels were assessed and correlated with tumor vs. normal and clinicopathological features (i.e., low Gleason score < 7 and high Gleason Score ≥ 7) in a large cohort of human prostate normal (*n* = 52) and tumor tissues (*n* = 490) in TCGA PRAD from the Genomic Data Commons Data Portal, NIH (https://portal.gdc.cancer.gov/projects/TCGA-PRAD, accessed on 1 October 2022).

*ANLN* and *ECT2* expression levels were also assessed and correlated with tumor vs. non-malignant and clinicopathological features (i.e., low Gleason score < 7 and high Gleason Score ≥ 7), in an AA prostate contralateral benign-tumor-matched cohort. The cohort represented a cross-sectional study of 74 men (*n* = 74) who were consented at Chicago Veterans Affairs Center Urology Clinic between November 2013 and 2019 under approval by the Jesse Brown Veterans Affairs Institutional Review Board (STU 73437). Patient Exclusion Criteria: Patients were excluded if they met any of the following criteria: presented with clinical metastatic disease; undergoing active surveillance or other treatment options; had a history of end-stage renal failure, liver failure, or parathyroid disorders; and prior treatment with androgen deprivation therapy or pelvic radiotherapy. All prostatectomy specimens were graded and staged according to the 2014 Gleason grading system and guidelines. 

### 2.14. Statistical Analysis

RT-qPCR was performed for RNA-seq DEG validation. A student *t*-test was conducted to compare differential gene expression levels in response to 1α,25(OH)_2_D_3_ treatment using Graphpad Prism version 10.1.2 (324) with FDR *p*-value < 0.05 reported.

## 3. Results

### 3.1. Ancestry Genotyping Reveals Significant West African Ancestry in RC-77N/E Cell Line

One hundred and five AIMs were selected from a larger previously validated set of markers to define critical genome candidate regions and characterize samples from diverse population groups [25,26]. This subset of AIMs contains SNPs capable of estimating individual West African (WAA), Native American (NA), and European (EUR) genetic ancestry. In the present study, we genotyped the RC-77N/E cell line, utilizing the established 105 AIM panel to assess WAA estimations. RC-77N/E is an immortalized non-malignant prostate cell line that was developed from an AA PCa patient, and we confirmed the WAA proportion of this cell line was 88.9% [38] (Figure S1A).

### 3.2. Pathway Enrichment Analysis Reveals Repression of Actin Cytoskeleton and Actin-Related Signaling Pathways Involved in Prostate Carcinogenesis

Extensive experimental and pre-clinical studies characterizing vitamin D metabolites, such as 1α,25(OH)_2_D_3_, effectively demonstrated the potential regulation of essential pathways involved in cellular maintenance and inhibiting carcinogenesis in a myriad of cell types [17]. To determine the effects of 1α,25(OH)_2_D_3_ treatment in AA non-malignant cells, we completed RNA sequencing analysis comparing 1α,25(OH)_2_D_3_-treated versus untreated replicates of the RC-77N/E non-malignant prostate cell line (*n* = 2, *n* = 3, respectively) (Figure S1A). A total of 1601 genes were identified as significantly differentially expressed in treated relative to untreated cells (499 upregulated and 1102 downregulated) (Figure S1B–D, Table S1).

**Figure 1:**
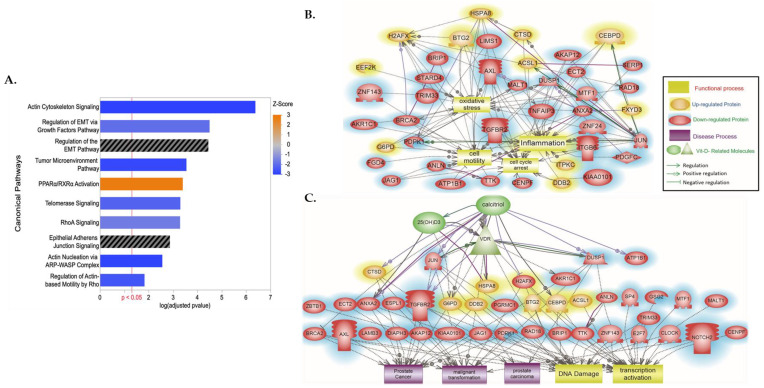
IPA and Pathway Studio Analysis of the DEGs between 1α,25(OH)_2_D_3_-treated and untreated AA non-malignant prostate cells. (**A**) Top significant canonical pathways and (**B**,**C**) disease and function terms enriched from 1601 DEGs between 1α,25(OH)_2_D_3_-treated and untreated AA non-malignant prostate cells. In figure (**A**), Z-score represents predicted activation (Z > 0, orange) or inhibition (Z < 0, blue), and bars in black have no predicted effect. *p*-value represents B–H adjusted *p*-value. In figure (**B**,**C**), proteins in an oval red color are downregulated, while those in an oval yellow color are upregulated, and their relationships with (**B**) oxidative stress, inflammation, cell motility, and cell cycle and (**C**) PCa, malignant transformation, prostate adenoma, DNA damage, and transcription activation are indicated. The raw data with the *p*-values of the relationship and pathways indicated in each of the generated panels are provided as supplementary raw data (Tables S3 and S4).

Ingenuity Pathway Enrichment Analysis of our top canonical pathways revealed significant enrichment and predicted repression of the following pathways: actin cytoskeleton signaling, regulation of Epithelial–Mesenchymal Transitioning (EMT) growth factors pathway, tumor microenvironment pathway, telomerase signaling, RhoA (Ras homology family member A) signaling, and actin nucleation via ARP-WASP (Actin-related Protein Wiskott–Aldrich Syndrome Protein) complex and regulation of actin-based motility by Rho (Figure 1A). Though identified as being significantly enriched, there was no predicted effect in the regulation of the EMT and adherens junction signaling pathway (*p*-adjusted B–H q < 0.05, Z-score NaN) (Figure 1A, Table S2). Nonetheless, using Elsevier’s Pathway Studio and ResNet Pathways Studio Propriety database, we additionally assessed the enrichment of DEGs associated with specific disease and function categories. We observed calcitriol-induced regulation of DEGs to be significantly associated with oxidative stress, inflammation, cell motility, cell cycle arrest (Figure 1B, Table S3), PCa, malignant transformation, prostate adenoma, DNA damage, and transcription activation (Figure 1C, Table S4). Gene ontology analysis revealed significant enrichment of differentially expressed genes in cytoskeletal, cell junction, and microtubule cellular processes. Analysis of molecular components revealed significant enrichment in actin binding, while also revealing significant enrichment of differentially expressed genes involved in cell cycle, cell division, and mitotic biological processes (Figure S2) (*p*-adjusted B–H q < 0.05).

### 3.3. DEG Algorithm and Bioinformatic Analysis Revealed Potential Survival PCa Biomarkers That Harbor VDRE Motifs, ANLN and ECT2

**Figure 2:**
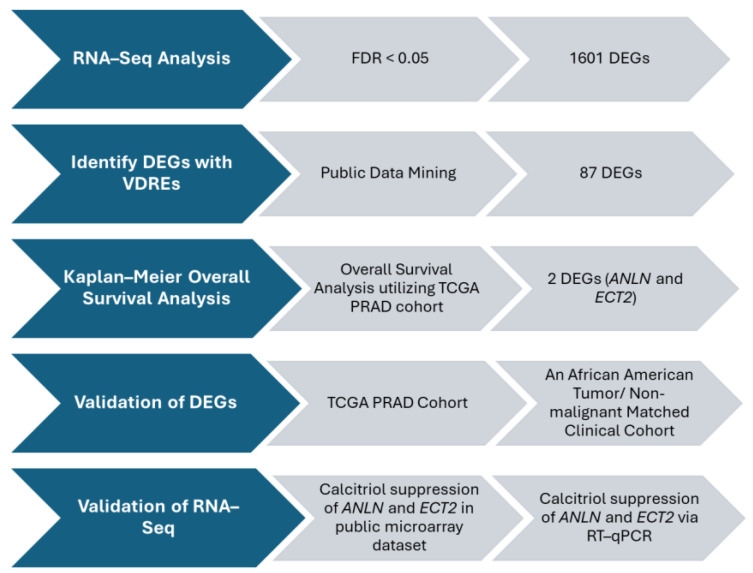
DEG Selection Algorithm. Selection algorithm designed to identify DEGs for validation and further downstream analyses.

Vitamin D_3_ and vitamin D metabolites, such as1α,25(OH)_2_D_3_, have been demonstrated to elicit pleiotropic anti-tumorigenic effects by binding to the VDRE motif in the promoter region of genes to initiate transcription and regulate expression [39]. To identify biomarkers that are regulated by 1,25(OH)_2_D_3_ and that may be essential to maintaining a non-malignant phenotype in AA prostate cells, a selection algorithm was designed and implemented (Figure 2). Data mining of large-scaled ChIP–Seq public datasets was performed to identify genes with putative VDREs whose transcripts were differentially regulated by vitamin D analogues (Table S5) [40,41]. A total of 87 DEGs were identified by comparison of their list of differentially expressed transcripts to our list of transcripts regulated by 1,25(OH)_2_D_3,_ at 24 h, in RC-77N/E cells (Table S5). Of the 87 DEGs with known VDREs, 41 genes were involved in canonical pathways that were significantly enriched with our DEGs associated with vitamin D treatment. We observed a significant enrichment of DEGs, with known VDREs, in the top 10 canonical pathways, which were mostly related to actin cytoskeleton signaling (Figure 1A, Table S6). Significant DEGs included *ANLN* (Anillin), *CKAP4* (Cytoskeleton-Associated protein 4), *DIAPH3* (Diaphanous-Related Formin 3), *FGF5* (Fibroblast Growth Factor 5), *ITGB6* (Integrin subunit Beta 6), and *PDGFC* (Platelet-Derived Growth Factor C). *PDGFC*, *FGF5*, and *TGFBR2* (Transforming Growth Factor Beta Receptor 2) were also involved in EMT-related pathways with *JAG1* (Jagged Canonical Notch Ligand 1); while, *JUN* (Jun proto-oncogene, *AP*-1 transcription factor subunit), *ECT2* (Epithelial Cell Transforming 2), and *NOTCH2* (Neurogenic locus notch homolog protein 2) are involved with the Epithelial Adherens Junction signaling pathway (Figure 1B,C, Table S6). In addition, *ECT2* was shown to directly regulate and inhibit cell cycle arrest and regulate malignant transformation, while *ANLN* was shown to regulate cell motility (Figure 1B,C, Tables S4 and S5).

**Figure 3:**
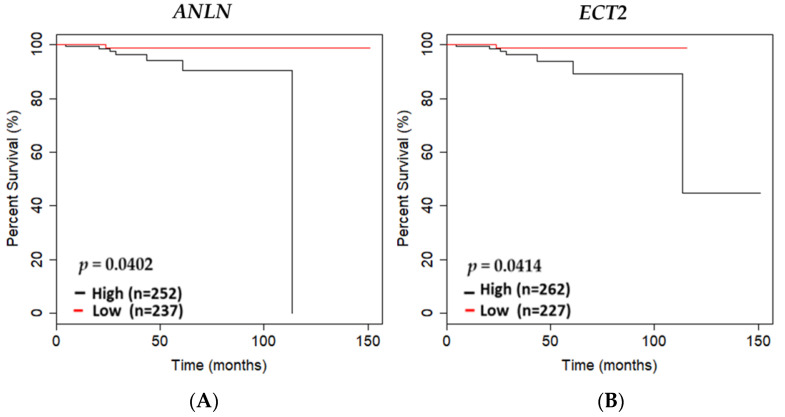
*ANLN* and *ECT2* expression association with overall survival in the TCGA PRAD cohort. Kaplan–Meier curves showing overall survival outcomes of (**A**) *ANLN* low- versus high-expressing tumors based on the mean of mRNA expression of specific gene of interest in the TCGA PRAD cohort; (**B**) *ECT2* low- versus high-expressing tumors based on the mean of mRNA expression of specific gene of interests in the TCGA PRAD cohort (*n* = 489). Low expression is shown in red, and high expression is shown in black. N values of high- versus low-expressing and *p*-values generated using student’s *t*-test are reported.

Utilization of the TCGA PRAD cohort (*n* = 490) was used to further screen DEGs that may be potential biomarkers essential for maintaining a non-malignant prostate phenotype (Table S5). Upon filtering DEGs, using the TCGA PRAD cohort and performing overall survival analysis, we revealed overexpression of *ANLN* and *ECT2* to be significantly associated with poor survival of PCa patients in TCGA PRAD cohort (*p* = 0.0402; *p* = 0.0414, respectively) (Figure 3A,B).

### 3.4. ANLN and ECT2 Overexpression Is Correlated with Tumorigenesis and Gleason Score in the TCGA PRAD Cohort and an AA Clinical Cohort

**Figure 4:**
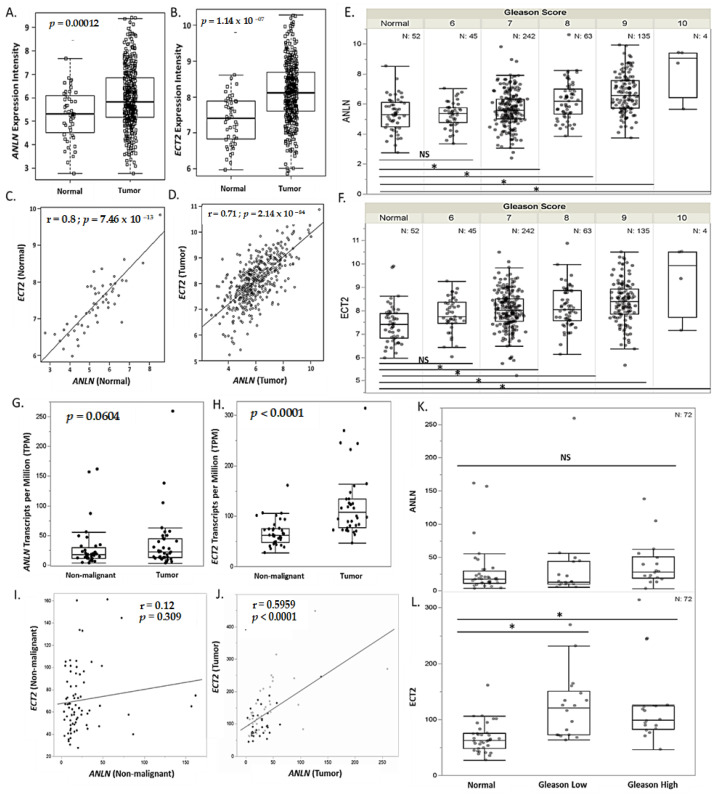
*ANLN* and *ECT2* are significantly correlated and more highly expressed in prostate tumor tissue compared to non-malignant tissue. (**A**,**B**) Overexpression of *ANLN* and *ECT2* mRNA expression in tumor (*n* = 490) compared to normal (*n* = 52), measured in expression intensity. (**C**,**D**) Pearson correlation analysis of *ANLN* and *ECT2* expression in normal (*n* = 52) compared to tumor (*n* = 490), measured in expression intensity. (**E**,**F**) *ANLN* and *ECT2* association with sum Gleason score in the TCGA PRAD cohort (*p* < 0.05), measured in expression intensity. (**G**,**H**) Overexpression of *ANLN* and *ECT2* mRNA expression in our AA clinical match tumor (*n* = 73)/non-malignant (*n* = 74) cohort, measured in TPM. (**I**,**J**) Pearson correlation analysis of *ANLN* and *ECT2* expression in non-malignant (*n* = 73) and tumor-matched tissue (*n* = 73), measured in TPM. (**K**,**L**) *ANLN* and *ECT2* association with sum Gleason score in an AA clinical cohort (*p* < 0.05), measured in TPM. *p*-values and r-coefficients are reported on graph (**A**–**D**,**G**–**J**). * *p* < 0.05; NS = not significant (**E**,**F**,**K**,**L**).

Increased expression of *ANLN* and *ECT2* in tumors has been shown to have pro-tumorigenic effects [42,43,44,45]. Given our findings, which suggest that low expression of *ANLN* and *ECT2* are associated with increased overall survival, we validated *ANLN* and *ECT2* expression in both the TCGA PRAD cohort and an AA prostate contralateral benign-tumor-matched cohort. We assessed *ANLN* and *ECT2* expression differences in tumor versus non-malignant tissue. Here, we show *ANLN* and *ECT2* to be significantly overexpressed in tumors compared to normal in the TCGA PRAD cohort (*n* = 490) (*p* = 0.00012, *p* < 0.0001, respectively) (Figure 4A,B). *ANLN* and *ECT2* expression was shown to be strongly correlated in normal and tumors as well (Figure 4C,D). In addition, *ANLN* and *ECT2* were shown to be significantly associated with increased Gleason scores (high Gleason score ≥ 7) (*p* < 0.05) (Figure 4E,F). In our AA prostate contralateral benign-tumor-matched cohort (*n* = 73), *ANLN* was shown to be nominally overexpressed in tumors compared to non-malignant (*p* = 0.06), while *ECT2* was significantly overexpressed in tumor compared to non-malignant matched tissue (*p* < 0.00001) (Figure 4G,H, Table S7). We also report only *ECT2* to be significantly associated with increased Gleason scores (high Gleason score ≥ 7) in our AA clinical cohort (*p* < 0.05) (Figure 4L). However, *ANLN* and *ECT2* expressions were significantly correlated in tumors of the TCGA PRAD and AA clinical cohorts (Figure 4I,J). Thus, suggesting under-expression of *ANLN* and *ECT2* may be essential in maintaining a non-malignant phenotype in EA and AA PCa tissue.

### 3.5. ANLN and ECT2 Are Significantly Downregulated in 1α,25(OH)_2_D_3_-Treated EA and AA Non-Malignant Prostate Cell Lines

**Figure 5:**
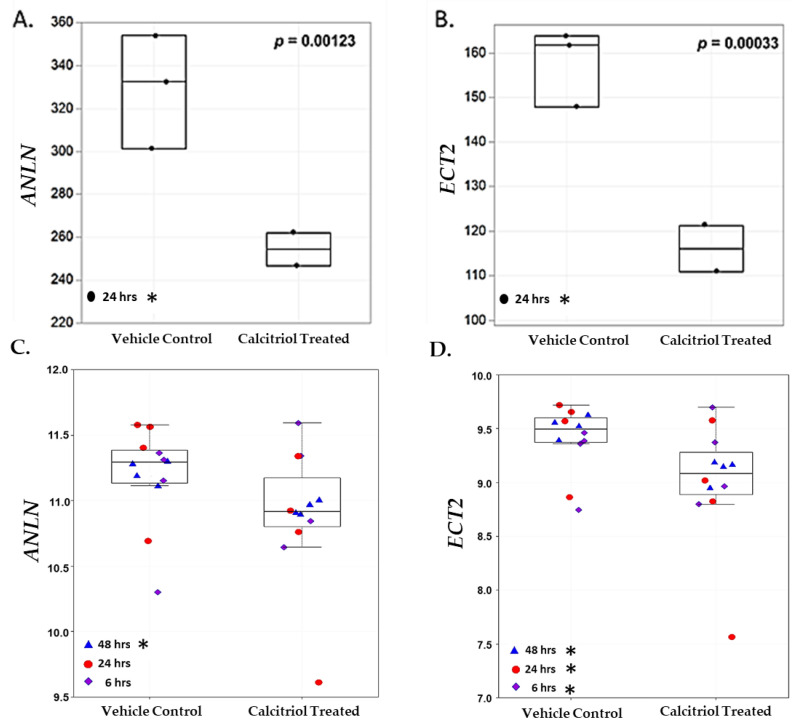
*ANLN* and *ECT2* are downregulated upon 1α,25(OH)_2_D_3_ treatment in RC-77N/E and RWPE1 cell lines. Boxplots of (**A**) *ANLN* and (**B**) *ECT2* gene expression among untreated controls (*n* = 3) and 1α,25(OH)_2_D_3_ treated (*n* = 2), at 24 h, in RC-77N/E cell line replicates. (**A**) *ANLN* and (**B**) *ECT2* are measured in TPM. In the validation microarray study, boxplots of (**C**) *ANLN* and (**D**) *ECT2* gene expression among untreated controls (*n* = 12) and 100 nM 1α,25(OH)_2_D_3_ (*n* = 12) at (*n* = 4) 6 h (blue triangle), 24 h (red circle), and 48 h (purple diamond); RWPE1 cell line replicates. (**C**) *ANLN* and (**D**) *ECT2* are measured in log2 fold change intensity. For boxplots, student’s *t*-test *p*-values are reported on the graphs. * FDR *p* < 0.05.

**Figure 6:**
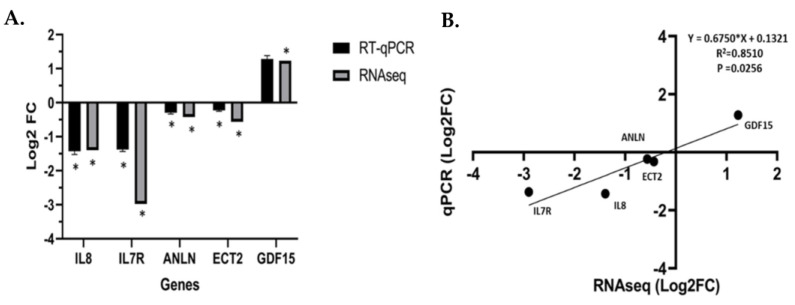
RT-qPCR validation of RNA–Seq analysis. (**A**) RT–qPCR confirmation results for the randomly relevant selected DEGs from the RC-77N/E-treated 1α,25(OH)_2_D_3_ compared to the untreated control. (**B**) Regression analysis of the log2 fold change values between the RNA–Seq and RT–qPCR validation of the RC-77N/E-treated 1α,25(OH)_2_D_3_ compared to the untreated control. The GAPDH gene was used as an internal reference control gene. RT–qPCR was performed with replicates, * FDR *p* < 0.05 is significant.

Vitamin D metabolite, 1α,25(OH)_2_D_3_, elicits anti-tumorigenic effects by regulating and targeting genes that harbor VDREs in or proximal to the promoter region. In our study, 1α,25(OH)_2_D_3_ significantly downregulated and suppressed *ANLN* and *ECT2* expression in an AA non-malignant prostate cell line RC-77N/E (*ANLN* log2 fold change = −0.422, FDR-adj *p* = 0.001; *ECT2* log2 fold change = −0.562, FDR-adj *p* = 0.0003, respectively) (Figure 5A,B, Table S1). RT-qPCR analysis was performed to validate RNA-Seq results of 1α,25(OH)_2_D_3_-mediated significant suppression of *ANLN* and *ECT2* expression in RC-77N/E (FDR-adj *p* < 0.05) (Figure 6A,B). To further validate 1α,25(OH)_2_D_3-_mediated suppression of *ANLN* and *ECT2* gene expression, we utilized published microarray data from the public Affymetrix U133 Plus 2.0 mRNA gene expression profile (GEO accession GSE15947) [46], which analyzed the effects of 1α,25(OH)_2_D_3_ on pathway signaling and DEGs in RWPE1, an EA non-tumorigenic prostate epithelial cell line [46]. RWPE1 cells were treated with 100 nM 1α,25(OH)_2_D_3_ at 6 h, 24 h, and 48 h time points, and post-treatments (four controls and four treated replicated) were assayed per time point. Kovalenko and colleagues showed *ANLN* and *ECT2* were downregulated upon 1α,25(OH)_2_D_3_ treatment (*ANLN* log2 fold change < −1, FDR-adj *p* < 0.05, at 24 h; *ECT2* log2 fold change < −1, FDR-adj *p* = 0.0282, for all time points) (Figure 5C,D), which corroborates and validates our findings of 1α,25(OH)_2_D_3_ significantly suppressing *ECT2* in the RC-77N/E cell line (*ANLN* log2 fold change = −0.422, FDR-adj *p* = 0.00123; *ECT2* log2 fold change = − 0.562, FDR-adj *p* = 0.00033).

### 3.6. ANLN and ECT2 Are Co-Expressed and 1α,25(OH)_2_D_3_-Mediated Suppression of ANLN and ECT2 Leads to Predicted Repression of Carcinogenesis

**Figure 7:**
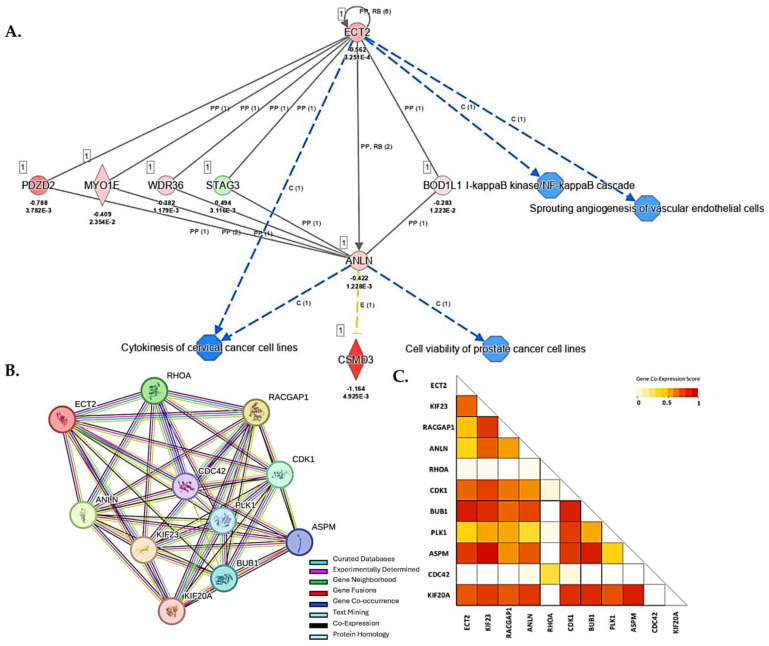
IPA and STRING PPI network analysis of DEGs associated with *ANLN* and *ECT2*. (**A**) 1α,25(OH)_2_D_3_ treatment led to predicted repression of molecules and disease/function categories up- and downstream of *ANLN* and *ECT2*. Red (downregulated), green (upregulated), blue (predicted inhibition), and yellow (inconclusive). Log2 fold change and adjusted *p*-values for DEGs are reported in figure. (**B**) *ANLN* and *ECT2* Protein–Protein Interaction Network analysis using STRING database. Legend reported in figure. (**C**) Co-expression scores based on RNA expression patterns and on protein co-regulation provided by STRING ProteomeHD. Co-expression scale reported in figure.

The pleiotropic effects of the vitamin D metabolite 1α,25(OH)_2_D_3_ may suppress PCa cellular dysfunctions by inhibition of cellular proliferation, cell cycle progression, cell invasiveness and angiogenesis, or by induction of apoptosis [47]. To explore potential vitamin D-mediated DEGs that act in concert with *ANLN* and *ECT2* and downstream biological processes that may be altered due to 1α,25(OH)_2_D_3_ treatment in prostate cell lines, we performed “de novo” IPA network and STRING protein–protein interaction (PPI) analysis. IPA network analysis allows for relevant networks to be identified based on extensive records maintained in the Ingenuity Pathways Knowledge Base [48]. Upon 1α,25(OH)_2_D_3_ treatment, the following DEGs were shown to act in concert with *ANLN* and *ECT2* and were found to be significantly downregulated: *PDZD2* (PDZ domain containing 2) (log2 fold change = − 0.788; FDR-adj *p* = 3.78 × 10^−3^), *MYO1E* (Myosin-1E) (log2 fold change = − 0.409; FDR-adj *p* = 2.35 × 10^−2^), and *WDR36* (WD Repeat Domain 36) (log2 fold change = − 0.382; FDR-adj *p* = 1.179 × 10^−3^). Upstream regulator *STAG3* (Stromal Antigen 3) (log2 fold change = 0.494; FDR-adj *p* = 3.1 × 10^−3^) was observed to be significantly upregulated. Downregulation of *ANLN* and *ECT2* led to predicted repression of cytokinesis of cervical cancer cell lines (*p* < 0.05). Predicted repression of cell viability of PCa cell lines was associated with *ANLN* repression, while repression of *NF*-kB (Nuclear Factor-kappa B) cascade and angiogenesis were also associated with downregulation of *ECT2* (*p* < 0.05) (Figure 7A). Furthermore, STRING analysis revealed a strong predictive functional interaction score of 0.994 between *ANLN* and *ECT2* (Table S10). Further investigation of proteins, in network with *ANLN* and *ECT2*, showed nine nodes (excluding *ANLN* and *ECT2*) with a protein–protein interaction (PPI) enrichment *p*-value of 4.57 × 10^−3^ involved in actin cytoskeleton remodeling, mitotic, and cell cycle regulation (FDR < 0.01, strength > 2.0) (Table S10). Our findings show two out of the nine genes, that are co-expressed and interact with *ANLN* and *ECT2*, were also significantly downregulated in our study. For example, *ASPM*—Assembly Factor for Spindle Microtubules (log2 fold change = − 0.373; FDR-adj *p* < 0.05)—and *CDK1*—Cyclin Dependent Kinase 1(log2 fold change = − 0.421; FDR-adj *p* < 0.05)—were significantly downregulated upon 1α,25(OH)_2_D_3_ treatment (Figure 7B,C).

## 4. Discussion

Established data have given rise to the postulate that elucidation of vitamin D_3_ alteration of cellular transcriptomic profiles is essential for identifying potential cancer biomarkers and pathway signaling cascades that can be targeted for cancer therapy [49]. However, due to the paucity of diverse prostate pre-clinical models available [50] and even fewer options that span pre-malignancy through various stages of disease manifestation [51], the pleiotropic anti-tumorigenic effects of vitamin D metabolite, 1α,25(OH)_2_D_3_, in normal prostate physiology remain unclear, especially for high-risk populations that may be innately vitamin D deficient (e.g., AA men). In our study, we performed RNA– Seq analysis and compared DEGs in 1α,25(OH)_2_D_3_-treated vs. non-treated AA non-malignant prostate cells to elucidate 1α,25(OH)_2_D_3_ regulation of pathways that underly prostate carcinogenesis and malignant transformation. In addition, we identified potential survival PCa biomarkers that may lead to developing novel interventions and better stratification of high-risk populations. Thus, we report 1α,25(OH)_2_D_3_ may repress processes that underly prostate carcinogenesis by regulating essential DEGs involved in mitotic cell division and cell motility mechanics driven by actin cytoskeleton signaling. We also reported that 1α,25(OH)_2_D_3_ suppresses potential survival PCa biomarkers, *ANLN* and *ECT2,* which are essential regulators of cytokinesis via the RhoA (Ras homology family member A) signaling pathway. The RhoA pathway is essential in every step of carcinogenesis and has been shown to tightly regulate mitotic cytokinesis and cell motility via actin cytoskeleton signaling. Therefore, our results indicate a possible mechanism where 1α,25(OH)_2_D_3_ may suppress the *ANLN–ECT2* complex to alter cytokinesis and RhoA signaling to inhibit prostate carcinogenesis. This underscores the potential critical role of vitamin D_3_ and vitamin D metabolites in the maintenance of prostate epithelial development and function in maintaining a non-malignant phenotype. 

*ANLN,* an actin-binding molecule, and *ECT2*, a guanine nucleotide exchange factor, are necessary for maintaining cytokinesis and cell cycle regulation [52]. During cytokinesis, at the onset of anaphase, *ECT2* is dephosphorylated, which allows it to bind to the centralspindlin, a major regulator of spindle assembly. The *ECT2*–centralspindlin then binds to *ANLN* to form a complex that activates the RhoA signaling pathway [45]. Upon activation of several downstream effectors, the sliding of myosin heads along the actin filaments is triggered leading to the formation and ingression of the cleavage furrow [45,52]. Dysfunction during the critical step of cytokinesis has been shown to lead to chromosomal instability (CIN), aneuploidy, and apoptosis [53]. *ANLN* and *ECT2* also play an essential role in modulating actin cytoskeletal signaling to facilitate efficient cell motility [45]. In prostate cells, rapid reorganization of actin filaments and suppression of actin organization signaling pathways have been reported to decrease invasion, migration, and cancer cell survival [54,55,56]. Interestingly, our pathway enrichment analysis revealed 1α,25(OH)_2_D_3_ treatment led to significant repression of DEGs involved in actin cytoskeleton signaling and actin-related signaling pathways, such as RhoA. This finding corroborates data that have implicated 1α,25(OH)_2_D_3_ in rapid cytoskeleton configuration changes in non-malignant and malignant cells [22,46,57]. 

In our study, we report that 1α,25(OH)_2_D_3_ significantly suppresses *ANLN* and *ECT2* expression in an AA non-malignant prostate cell line, and it is plausible that increasing 1α,25(OH)_2_D_3_ concentration may be directly proportionate to increased suppression. Kovalenko and colleagues corroborated our findings by demonstrating 100 nM of 1α,25(OH)_2_D_3_ at 6 h, 24 h, and 48 h significantly suppressed *ECT2* expression, while significant suppression of *ANLN* expression was observed at 48 h in a non-malignant EA prostate cell line [46]. Our data suggest 1α,25(OH)_2_D_3_ may regulate *ANLN* and *ECT2* through a novel VDR-mediated pathway, which significantly inhibits RhoA, and other actin-related signaling cascades, needed to facilitate cell cycle transitioning during mitosis in non-malignant and malignant prostate cells. Interestingly, *RhoA*, which is an androgen receptor targeted gene, has also been shown to be dysregulated and overexpressed in AA prostate tumors [58]. Thus, we suggest *ANLN* and *ECT2* as novel biomarkers that modulate RhoA signaling. Taken together, 1α,25(OH)_2_D_3_ suppression of *ANLN* and *ECT2,* which modulate RhoA signaling, may be essential for maintaining a non-malignant prostate phenotype, which implies a novel mechanism for chemoprevention therapeutic targeting, especially for PCa in AA men.

We also provide evidence that *ANLN* and *ECT2* may serve as prognostic survival PCa biomarkers to better stratify high-risk populations, especially in AA men with advanced PCa. Our analysis of overall survival in the TCGA PRAD database revealed that high expression of *ANLN* and *ECT2* was significantly associated with decreased survival, whereas low expression was associated with better overall survival of PCa patients. We validated *ANLN* and *ECT2* mRNA expression in tissue, utilizing the TCGA PRAD and an AA non-malignant/tumor-matched clinical cohort. *ANLN* and *ECT2* mRNA expression was shown to be significantly overexpressed in tumors and under-expressed in normal tissue (|log2 fold change| > 1, *p* < 0.05) in the TCGA PRAD cohort. In addition, *ANLN* and *ECT2* expression was strongly correlated in both normal and tumor tissues, which is to be expected due to *ECT2* forming a complex with *ANLN* to regulate cytokinesis [45]. In our AA clinical cohort, though *ANLN* was not significantly overexpressed in malignant tissue (*p* = 0.06), *ECT2* was significantly overexpressed in malignant compared to non-malignant matched tissue (*p* < 0. 0001). Additionally, *ANLN* and *ECT2* expression was weakly correlated in the non-malignant matched tissue; however, we detected strong positive correlations in malignant matched tissue. This suggests *ANLN* and *ECT2* may be regulating pathways independently to maintain a non-malignant phenotype, but the *ANLN*–*ECT2* complex may be essential during prostate carcinogenesis in AA PCa tumors. Taken together, our data suggest that low and/or suppressed expression of *ANLN* and *ECT2* may increase survival of PCa patients, which may prove to be beneficial when stratifying populations with advanced PCa for treatment. 

The potential prognostic survival benefits of *ANLN* and *ECT2* reported in our study are supported by Zhang and colleagues’ evaluation of the carcinogenic roles of *ANLN* in various types of cancers via a pan-cancer analysis utilizing the TCGA-GTEx database. They reported *ANLN* expression was associated with patients’ progression-free intervals (PFIs), while *ANLN* overexpression significantly was associated with patient disease-free intervals (DFIs) (HR = 1.06, *p* < 0.0001) in the TCGA PRAD cohort [59]. Thus, this finding implicates *ANLN* as a promising biomarker pivotal in influencing tumor progression and development [60,61]. Guo and colleagues demonstrated that immunohistochemical staining for *ECT2* on a human tissue microarray was positively associated with clinical pathological parameters such as Gleason score, pathological grade, clinical stage, tumor invasion, lymph node involvement, and distant PCa metastasis. Their immunohistochemical results showed that the expression levels of *ECT2* protein were enhanced in EA PCa tissues [62]. In our study, we validated *ANLN* and *ECT2* expression in a separate independent clinical cohort within a high-risk population and reported *ECT2* expression is significantly overexpressed in tumors compared to non-malignant tissue and is positively associated with clinicopathological features (i.e., Gleason score) in AA men with PCa. 

Studies investigating the role of *ANLN* and *ECT2* in prostate carcinogenesis are diminutive. Previous studies report that the knockdown of *ANLN* and *ECT2* inhibits cell motility, cellular growth, migration, and invasion [42,63,64,65,66]; while overexpression of *ECT2*, in vivo, has been shown to have anti-apoptotic effects [42]. Given that *ANLN* and *ECT2* have been demonstrated to tightly regulate cytokinesis and actin organization via the RhoA/ROCK pathway [45], the potential of targeting the *ANLN, ECT2,* and/or the *ANLN–ECT2* complex in patients with advanced PCa warrants further investigation. It is plausible that 1α,25(OH)_2_D_3_-mediated suppression of tumor-specific actin-binding molecules (i.e., *ANLN* and *ECT2*) could be utilized in combination with FDA-approved chemotherapeutics that target actin cytoskeletal dynamics and RhoA signaling to improve treatment response outcomes in PCa patients. Thus, our novel findings lay the foundation to further elucidate the anti-proliferative effects of 1α,25(OH)_2_D_3_ in non-malignant prostate maintenance and prostate carcinogenesis by targeting potential survival PCa biomarkers, *ANLN* and *ECT2*. 

## 5. Conclusions

In this study, we rigorously analyzed public data and performed separate independent experiments to validate and to support the significance of our findings. Our findings suggest 1α,25(OH)_2_D_3_ targets and suppresses survival PCa biomarkers (*ANLN* and *ECT2*) in a non-malignant AA prostate cell line. Given the established pleiotropic effects of vitamin D_3_ treatment (i.e., anti-proliferative, anti-inflammatory, and immune modulatory impact on cellular structural mechanics), as well as effects on the regulation of potential survival biomarkers (e.g., *ANLN* and *ECT2*) associated with metastasis, there is an incentive to investigate vitamin D_3_ as a targeted therapeutic option optimized for AA men with localized PCa or as a primary chemoprevention.

## 6. Limitations

The empirical results reported herein should be considered in the light of some limitations. It is important to note that we observed calcitriol significant suppression of potential survival PCa biomarkers, *ANLN* and *ECT2*, in a non-malignant AA prostate cell line. Given that our data suggests *ANLN* and *ECT2* are essential to prostate carcinogenesis, further investigation of calcitriol regulation of these DEGs in AA PCa and metastatic cell lines is needed. In addition, investigation of the role of this novel ANLN/ECT2 signaling axis in an in vivo mice model would be valuable to validating *ANLN* and *ECT2* as potential drug targets for therapeutics. 

## Data Availability

The RNA–Seq dataset can be accessed via NCBI Gene Expression Omnibus (GEO; https://www.ncbi.nlm.nih.gov/geo/ (submitted on 13 May 2024) under accession number GSE267396. In addition, data analyzed during the current study is available from the corresponding author upon reasonable request.

## Supplementary Materials

The following supporting information can be downloaded at https://www.mdpi.com/article/10.3390/biology13050346/s1. Figure S1: DEG analysis of RC-77N/E cell line replicates; Figure S2: GO analysis of associated DEGs in RC-77N/E; Table S1: RNA-Seq analysis of RC-77N/E 1601 DEGs; Table S2: IPA Top20 canonical pathways; Table S3: Pathway Studios gene–gene interactions impacting biological processes; Table S4: Pathway Studios gene–gene interaction association with disease and function; Table S5: A total of 87 DEGs with known VDREs and OSKM data; Table S6: VDRE DEGs Top20 canonical pathways; Table S7: *ANLN*–*ECT2* expression in PCa cohort; Table S8: STRING analysis; Table S9: STRING PPI *ECT2* and *ANLN*; Table S10: STRING network statistics.

## Abbreviations

PCa	Prostate Cancer
AA	African American
EA	European American
RNA	Ribonucleic Acid
RNA-Seq	Ribonucleic Acid Sequencing
RT-qPCR	Real-Time Quantitative Polymerase Chain Reaction
PCR	Polymerase Chain Reaction
IPA	Ingenuity Pathway Analysis
FDR	False Discover Rate
EMT	Epithelial–Mesenchymal Transition
RhoA	Ras Homology Family Member A
DEG	Differentially Expressed Gene
VDRE	Vitamin D Response Element
TCGA	The Cancer Genome Atlas
PRAD	Prostate Adenocarcinoma
ANLN	Anillin
ECT2	Epithelial Cell Transforming 2
1α,25(OH)2D3	Calcitriol or 1,25-dihydroxyvitamin D3
PSA	Prostate-Specific Antigen
UV	Ultraviolet
25 hydroxyvitamin D3	Calcidiol
c-MYC	Cellular Myelocytomatosis Oncogene
G0/G1 phase	Growth 0/Growth 1 phase
ECM	Extracellular Matrix
TME	Tumor Microenvironment
AIM	Ancestral Informative Marker
WA	West African
NA	Native American
WAA	West African Ancestry
SNP	Single Nucleotide Polymorphism
DNA	Deoxyribonucleic Acid
CHIP	Chromatin Immunoprecipitation
MALDI-TOF	Matrix-Assisted Laser Desorption/Ionization Time of Flight
ATCC	American Type Culture Collection
OD	Optical Density
PCA	Principal Component Analysis
GO	Gene Ontology
cDNA	Complimentary DNA
GAPDH	Glyceraldehyde 3-Phosphate Dehydrogenase
GEO	Gene Expression Omnibus
JUN	Jun Proto-oncogene AP-1 transcription factor subunit
JUNB	JunB Proto-oncogene AP-1 transcription factor subunit
EGFR	Epithelial Growth Factor Receptor)
EGR	Early Growth Response
FOXM1	Forkhead box protein M1
IL17A	Interleukin 17A
IL1A	Interleukin1A
IL1B	Interleukin 1B
TNF	Tumor Necrosis Factor
ARP-WASP	Actin-related Protein Wiskott–Aldrich Syndrome Protein
CKAP4	Cytoskeleton-Associated Protein 4
DIAPH3	Diaphanous-Related Formin 3
FGF5	Fibroblast Growth Factor 5
ITGB6	Integrin Subunit Beta 6
PDGFC	Platelet-Derived Growth Factor
TGFBR2	Transforming Growth Factor Beta Receptor 2
JAG1	Jagged Canonical Notch Ligand 1
NOTCH2	Neurogenic Locus Notch Homolog Protein 2
PDZD2	PDZ Domain Containing 2
MYO1E	Myosin-1E
WDR36	WD Repeat Domain 36
STAG3	Stromal Antigen 3
NF-kB	Nuclear Factor-Kappa B
IGFBP-3	Insulin-like Growth Factor Binding Protein 3
FAS	Fas Cell Surface Death Receptor
Wee1	Wee1 G2 Checkpoint Kinase
G1/S phase	Growth 1/Synthesis Phase
G2/M phase	Growth 2/Mitosis Phase
CIN	Chromosome Instability
JAG1	Jagged 1
VDR	Vitamin D Receptor
