# Supplementary material for: 1,25-Dihydroxyvitamin D3 Suppresses Prognostic Survival Biomarkers Associated with Cell Cycle and Actin Organization in a Non-Malignant African American Prostate Cell Line
Source: Biology (Basel). 2024 May 15;13(5):346. doi: 10.3390/biology13050346 (PMC11118023; doi:10.3390/biology13050346)
Supplement: Supplementary file 1 [file biology-13-00346-s001.zip › SF1_DEG analysis of RC-77NE cell line replicates.pdf]

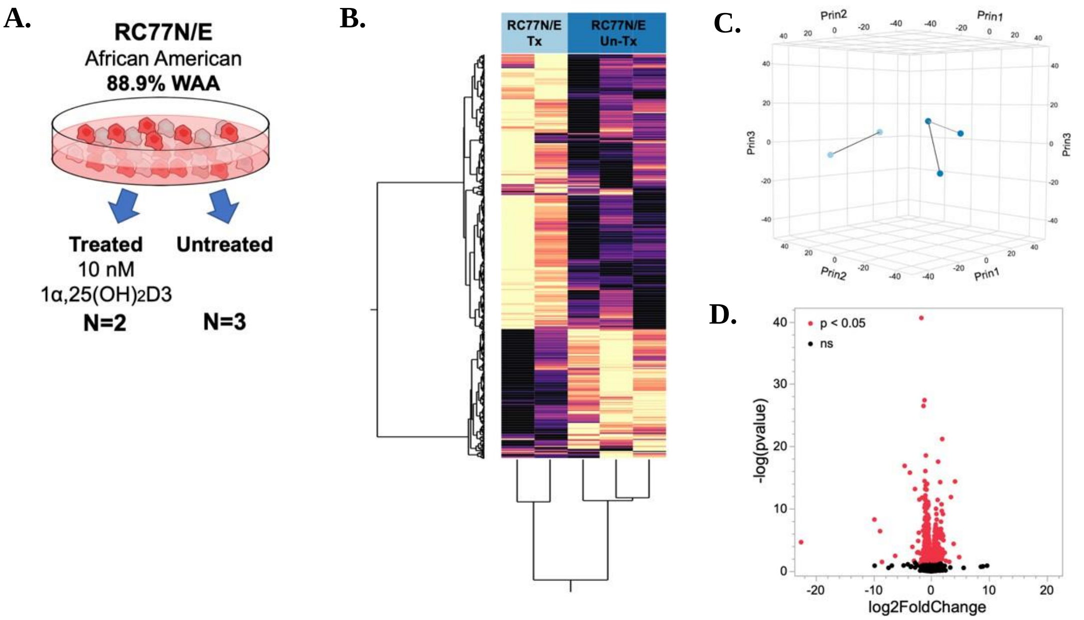

Supplemental Figure S1. Differential gene expression analysis of $1\alpha,25(OH)_2D_3$ treated versus untreated RC-77N/E cell line replicates. (A) Schematic of experimental design of Vitamin D treatment (10 nM $1\alpha,25(OH)_2D_3$) compared to untreated control replicates in the RC-77N/E African American prostate non-malignant cell line representing ~89% West African ancestry. (B) Unsupervised hierarchical clustering of 1601 differentially expressed genes (DEGs) between RC-77N/E treated (10 nM $1\alpha,25(OH)_2D_3$) vs untreated cells (padj < 0.05). Rows represent genes, columns represent cell line replicates. Light blue annotated columns represent RC-77N/E treated replicates, and dark blue annotated columns represent RC-77N/E untreated replicates. (C) 3D Principal Component Analysis (PCA) plot of RC-77N/E cell line replicates of untreated (dark blue) and treated (light blue) samples. (D) Volcano plot showing the distribution of DEGs between RC-77N/E treated (10 nM $1\alpha,25(OH)_2D_3$) vs untreated cells. Dots in red indicate adjusted p value of < 0.05 (**Red**).
