# Supplementary material for: 1,25-Dihydroxyvitamin D3 Suppresses Prognostic Survival Biomarkers Associated with Cell Cycle and Actin Organization in a Non-Malignant African American Prostate Cell Line
Source: Biology (Basel). 2024 May 15;13(5):346. doi: 10.3390/biology13050346 (PMC11118023; doi:10.3390/biology13050346)
Supplement: Supplementary file 1 [file biology-13-00346-s001.zip › SF2_GO analysis of associated DEGs in RC-77NE.pdf]

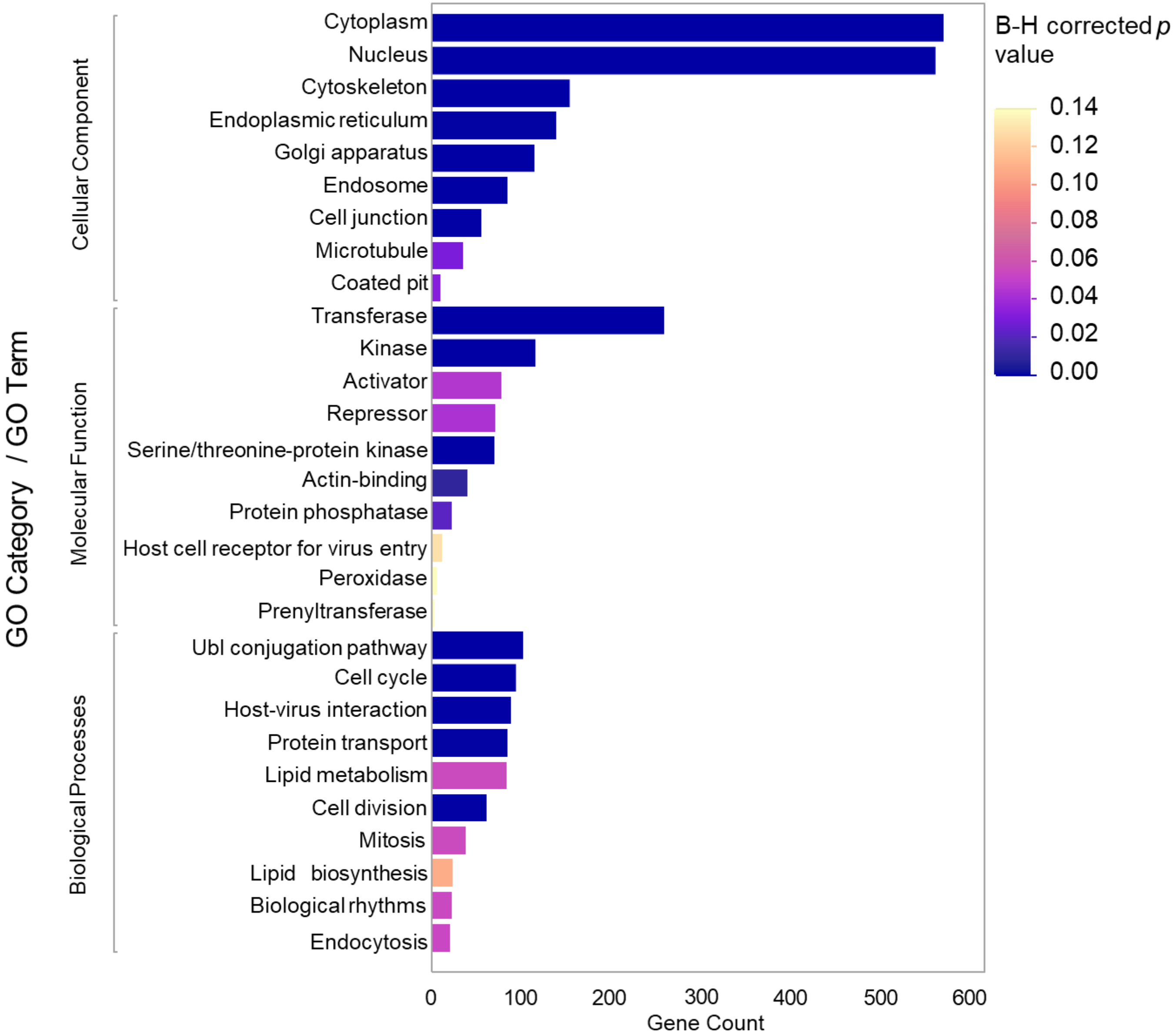

Supplemental Figure S2. Gene Ontology (GO) analysis of RC-77N/E 1α,25(OH)$_2$D$_3$ treatment associated DEGs. GO enrichment analysis of differentially expressed genes. Size of bar represents number of genes associated with a given GO term. Color scale indicates B-H corrected p <0.05 significant.
